# Efficacy of prophylactic methylprednisolone on reducing the risk of post-extubation stridor in patients after an emergency intubation: study protocol for a randomized controlled trial

**DOI:** 10.1186/s13063-020-04994-9

**Published:** 2021-01-06

**Authors:** Jingyi Wang, Joseph Harold Walline, Lu Yin, Yili Dai, Jiayuan Dai, Huadong Zhu, Xuezhong Yu, Jun Xu

**Affiliations:** 1 Emergency Department, State Key Laboratory of Complex Severe and Rare Diseases, Peking Union Medical College Hospital, Chinese Academy of Medical Science and Peking Union Medical College, Beijing, China; 2 Accident and Emergency Medicine Academic Unit, Prince of Wales Hospital, The Chinese University of Hong Kong, Hong Kong, China

**Keywords:** Post-extubation stridor, Corticosteroid, Emergency airway management, Multicenter, Randomized controlled trial

## Abstract

**Background:**

Post-extubation stridor (PES) is one of the most common complications of invasive respiratory support, with severe cases leading to possible extubation failure (reintubation within 48 h) and increased mortality. Previous studies confirmed that prophylactic corticosteroids play an important role in reducing the risk of PES and extubation failure. However, few studies have looked at the efficacy of corticosteroids on preventing PES in patients after an emergency intubation.

**Aim:**

To evaluate whether a single dose of methylprednisolone given over a set timeframe before extubation is effective in preventing PES in patients after an emergency intubation.

**Methods:**

A multicenter, randomized, placebo-controlled trial will be performed in an emergency department (ED) setting. The trial will include 132 patients who fail a cuff-leak test (CLT) prior to the intervention. Patients will be randomly assigned to either intravenous methylprednisolone (40 mg) or placebo 4 h prior to extubation. Other eligible patients who pass the CLT will be included in a non-intervention (observation) group. The primary endpoint is the incidence of PES within 48 h after extubation. Secondary endpoints include oxygen therapy, respiratory support requirements, reintubation secondary to PES, adverse effects within 48 h after extubation, hospital length of stay, and hospital mortality.

**Discussion:**

Patients who are intubated on an emergency basis have a higher risk of intubation-related complications. Previous studies have examined treatment regimens involving more than 10 different variations on corticosteroid treatments for PES prevention, while for ED therapy, only a simple and effective treatment would be appropriate. Corticosteroid administration is usually accompanied by adverse effects; thus, this study will be important for further risk stratification among intubated ED patients.

**Trial registration:**

Chictr.org.cn ChiCTR2000030349. Registered on 29 February 2020.

**Supplementary Information:**

The online version contains supplementary material available at 10.1186/s13063-020-04994-9.

## Background

Endotracheal intubation with mechanical ventilation is a common life support method in emergency resuscitation. However, intubation/extubation and invasive mechanical ventilation may lead to the development of complications such as post-extubation stridor (PES) or extubation failure (defined as reintubation within 48 h). Both of these complications are associated with a higher risk of ventilator associated pneumonia, prolonged hospital length of stay and increased mortality [1].

Corticosteroids have a broad anti-inflammatory effect which is well described in previous studies [2, 3]. They also have a vasopressor effect that contributes to reduced airway edema and less microvascular fluid leakage [4], which may help prevent laryngeal edema and PES. While a large body of evidence has revealed that prophylactic corticosteroids effectively reduce the incidence of PES and reintubation among high-risk patients [5], few studies have evaluated their effectiveness in patients after an emergency intubation. Due to the large ED population and impatient ward shortage, many patients are both intubated and extubated while remaining in the ED setting. Comparing with the previous studies developed in intensive care units (ICUs) [6], Shinohara [7] found the incidence of PES was higher in patients intubated on an emergency basis. This may due to multiple factors, including lack of familiarity with a patient’s history, limited time for airway evaluation and management, contraindications to many sedative agents due to an unstable clinical condition (e.g., hypotension or altered mental status), massive intravenous fluid therapy, or patient-endotracheal tube size mismatch.

Ideally, patients at high risk for PES should be identified before extubation. The cuff-leak test (CLT), defined as the difference in the actual tidal volume before and after cuff deflation, has been proposed for this purpose [8, 9]. Previous studies described a cuff leak volume less than 110 ml or 24% of tidal volumes as insufficient [10–12], and evidence showed absent or insufficient cuff leak volume suggested an increased risk of PES and extubation failure [8]. Several studies showed the CLT has a good specificity but poor sensitivity, and the accuracy for predicting PES varied with different studies [13–15]. Moreover, the measured tidal volume could also be influenced by other factors such as system compliance or airflow resistance [13]. However, the CLT is still preferred as it has been showed to decrease the risk of PES and reintubation [9].

We conceived the present protocol in order to evaluate whether a single dose of methylprednisolone given over a set timeframe before extubation is effective in reducing the incidence of PES in patients who had been intubated on an emergency basis and were approaching extubation. We hope to answer this question through a multicenter, double-blind, randomized controlled trial.

## Methods

### Trial design and setting

**Fig. 1:**
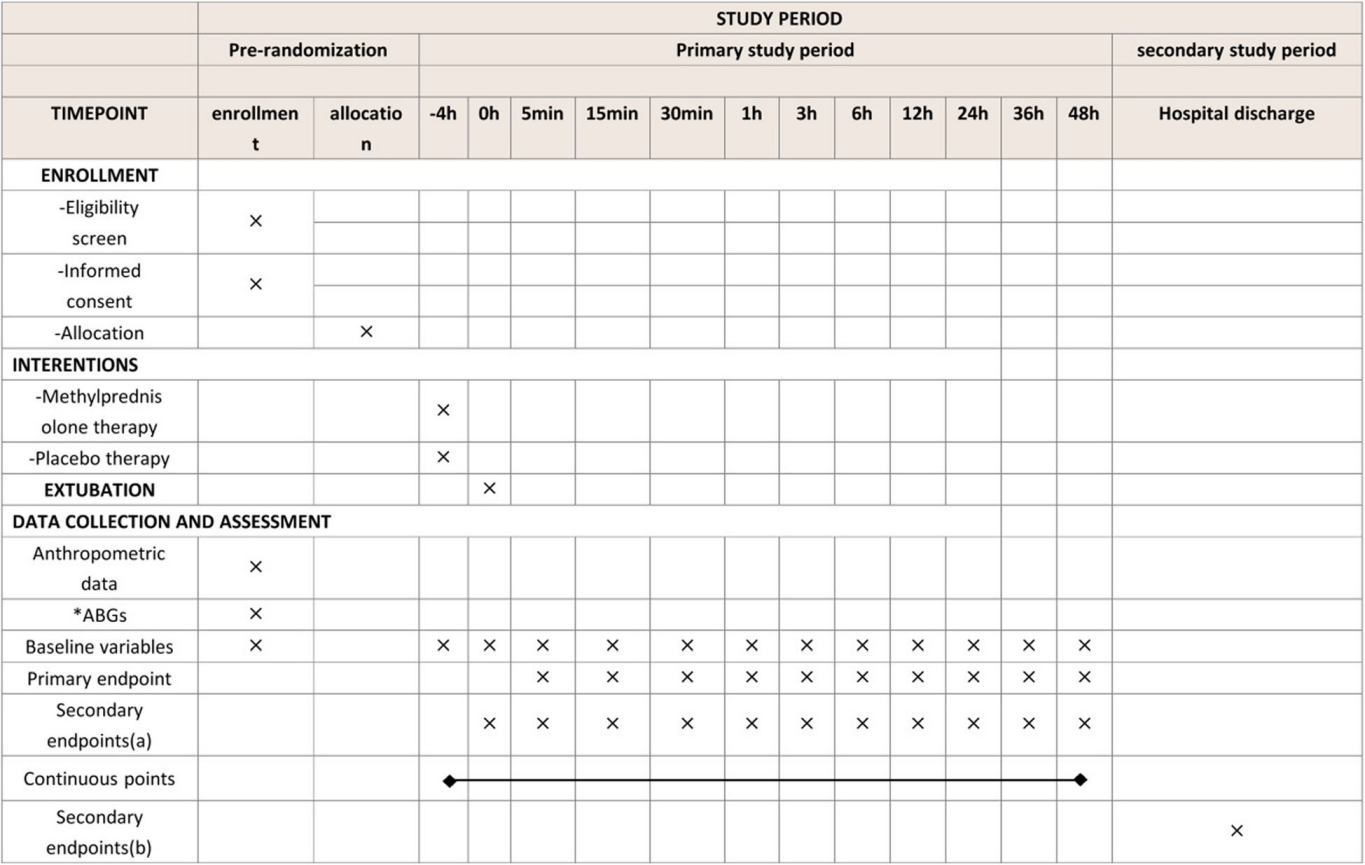
Standard Protocol Items: Recommendations for Interventional Trials (SPIRIT) schedule of enrolment, intervention, and assessments. Anthropometric data: age, gender, body mass index (BMI). Asterisk indicates the following: ABGs, collected for necessity only. Baseline variables: GCS score, APACHE II score, vital signs. Primary endpoint: occurrence of PES. Secondary endpoints (a): post-extubation oxygen therapy, respiratory support requirement, reintubation secondary to PES, adverse effects. Secondary endpoints (b): hospital length of stay and hospital mortality. Continuous points: SaO_2_ and PETCO_2_

This is an investigator-initiated, multicenter, superiority, randomized controlled trial that will include 132 participants in intervention arm. Six large tertiary hospitals in China will be involved in this trial. This study was prospectively registered with Chictr.org.cn on February 29, 2020 (identifier: ChiCTR 2000030349). The Ethics Committee of Peking Union Medical College Hospital, Chinese Academy of Medical Science, and Peking Union Medical College in Beijing has approved the trial protocol. The schedule of enrollment, intervention, data collection, and assessment follows the Standardized Protocol Items: Recommendations for Interventional Trials (SPIRIT) guidelines (see Fig. 1 for checklist and Additional File 1 for more details).

### Study population

Patients will be eligible for inclusion if they are aged 18–80 years, admitted into the emergency department (ED), and intubated emergently for one of the following conditions: (1) severe traumatic injury, (2) Glasgow Coma Scale (GCS) score < 8, (3) hypoventilation, (4) persistent hypoxemia (SaO_2_ ≤ 90%) despite supplemental oxygen, (5) persistent hypotension despite vasopressor treatment, or (6) cardiac arrest. Since most participants in our study will have no capacity to make decisions, written informed consent will be acquired from each patient’s next of kin prior to inclusion. Patients or their next of kin will be informed of the purpose, procedures, potential risks, and benefits of the study. Participants will be allowed to withdraw from the trial at any time without consequence.

Patients will be excluded if they meet at least one of the following criteria: pregnant or breastfeeding, chronically treated with corticosteroids or other anti-inflammatory drugs, extubated for patient comfort or on family request, unplanned or self-extubation, vocal cord dysfunction, deep sedation (defined as a Richmond Agitation-Sedation Scale (RASS) score ≤ − 4), or those diagnosed with gastrointestinal hemorrhage within the past 3 months.

There will be a research coordinator at each hospital to promote and monitor the trial.

### Intervention, control, and observation groups

Once eligible patients successfully pass a spontaneous breathing trial (SBT), a CLT will be performed to evaluate their risk for developing PES. Patients who had a cuff-leak volume (CLV) less than 24% of vital volume will be included in the intervention arm, where they will be randomly allocated to either the corticosteroid group or the placebo group. The planned extubation will be implemented within 6 h after allocation. Patients in the corticosteroid group and placebo group will be treated with an intravenous injection of methylprednisolone 40 mg (corticosteroid) or an equivalent volume of isotonic saline (placebo) 4 h prior to extubation. Patients with a CLV > 24% of tidal volume will serve as a control group, where extubation will be performed within 2 h after CLT completion.

All other standard precautions or treatments will be implemented in all patients.

Precautions include gentle extubation to avoid laryngotracheal mucosal injury, strict endotracheal tube (EET) fixation to avoid repeated friction between the EET and laryngotracheal mucosa, and EET nursing to reduce contamination risk. Any treatments for PES will be applied in accordance with each patient’s condition, including oxygen therapy with inhaled corticosteroid for mild airway edema and intravenous administration of hydrocortisone (100 mg) for moderate airway edema. Non-invasive ventilation or re-intubation will be carried out as clinically indicated if the above treatments are ineffective.

### Study endpoints

**Fig. 2:**
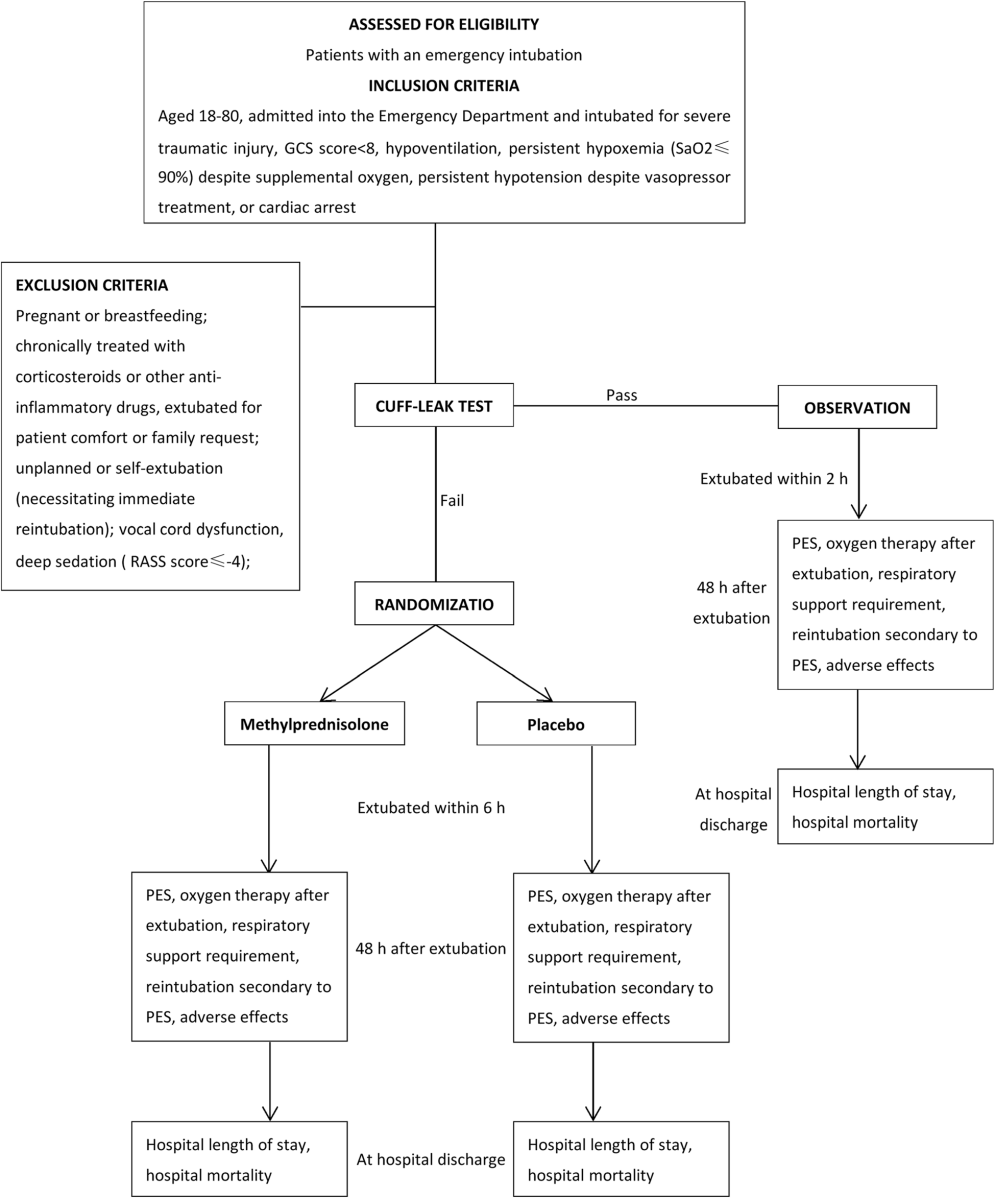
Study flow chart

The primary endpoint is the occurrence of PES within the first 48 h after extubation. Secondary endpoints include oxygen therapy after extubation, respiratory support requirements, reintubation secondary to PES within 48 h after extubation, and adverse effects (glycemic change, gastrointestinal bleeding or neuropsychiatric events). Clinical assessments will be done at 5, 15, 30, and 60 min, and then at 3, 6, 12, 24, 36, and 48 h after extubation. Hospital length of stay and hospital mortality will also serve as secondary endpoints (see Fig. 2 for study flow chart).

### Sample size calculation

We used a chi-squared statistical analysis of minimum sample size required to evaluate the effect of corticosteroids on our study outcomes. Cheng [10] showed that either a single or multiple doses of methylprednisolone 6 h before extubation reduced the incidence of PES compared to placebo (11.6%/7.1% vs 30.2%). Cheng [11] later reported that a single dose of methylprednisolone 4 h before extubation significantly reduced the incidence of PES from 39.4 to 15.8%. Other studies treating with prophylactic dexamethasone showed that it decreased the incidence of PES from 27.5–30.2 to 8.8–11.5% [12, 16]. We therefore anticipate an incidence of PES of 10% in the treatment group and 30% in the placebo group. With an 80% power to detect a difference and a two-sided alpha of 0.05, we anticipate needing 59 subjects in each group. Assuming a drop-out rate of 10%, we calculate a total minimum sample size of 132 subjects.

### Randomization and blinding

As female sex has been reported to be an independent risk factor for stridor and extubation failure [5, 7, 10, 17, 18], a gender-stratified block randomization will be performed in a 1:1 ratio. Within each stratum, a random block size of four will be used. After completion of the pre-extubation assessment and obtaining written informed consent, participants will be randomly assigned to either the intervention group or the control group. Staff not assigned to patient therapy or assessment will be responsible for injecting the intervention; thus, both patients and therapists involved will be blinded to treatment allocation. All analyses will be performed on an intention-to-treat basis.

### Data collection and management

For each participant, anthropometric data (gender, age, body mass index (BMI)) and baseline characteristics such as GCS score, Acute Physiology and Chronic Health II (APACHE II) score, vital signs (blood pressure, heart rate, respiratory rate, blood oxygen saturation and temperature), and arterial blood gases (ABGs) will be recorded as long as the patients are in-hospital. Arterial blood oxygen saturation (SaO_2_), as well as end tidal carbon dioxide (PETCO_2_) if available, will be continuously recorded. In order not to cause unnecessary pain to patients, ABGs will be collected when deemed clinically necessary during follow-up, while other baseline characteristics will be recorded at set times.

We will also record incidence of the following for each enrolled patient:

1. PES, defined as a presence of an audible high-pitched sound with respiration
2. Oxygen therapy after extubation (e.g., nasal catheter, reservoir mask or venturi mask)
3. Requiring a respiratory support, as defined by:
  - Presence of respiratory acidosis (an arterial pH of less than 7.35 with a partial pressure of arterial carbon dioxide of more than 45 mmHg)
  - Clinical signs of increased respiratory effort (use of accessory muscles, intercostal retractions, or paradoxical motion of the abdomen)
  - Respiratory rate > 30 breaths/min for two consecutive hours
  - Hypoxemia (SaO_2_ of < 90% with an FiO_2_ > 50%)
4. Reintubation secondary to PES
5. Adverse effects due to corticosteroids
6. Hospital length of stay
7. Hospital mortality.

Clinical data will be collected locally via the Research Electronic Data Capture (REDCap) system, an Internet-based electronic case report form (CRF). The research coordinators at each hospital will form a steering committee, which provides training and reviews study processes, to improve adherence to the protocol and resolve problems. In addition, they will regularly audit CRFs and contact responsible medical staff members every 3 months to ensure data quality and accuracy. All data use will be limited to study analysis only, and no individuals’ personal information will be published. Data confidentiality will be under the supervision of the study coordinators and the Ethics Committee of Peking Union Medical College Hospital.

### Statistical methods

The primary endpoint will be analyzed on an intention-to-treat basis, regardless of whether subjects complete their originally allocated treatment study protocol. Any reasons for protocol violations will be recorded and described. All *p* values will be two-tailed, and significance will be a *p* value < 0.05. Data will be presented as frequencies and percentages for categorical variables. Continuous variables will be expressed as means with standard deviations (when normally distributed) or as medians with interquartile ranges (for skewed distribution). Student’s *t* test (normal distribution) or Mann-Whitney *U* test (skewed distribution) will be used for group comparisons. Categorical variables will be compared using Pearson’s chi-squared test or Fisher’s exact test as appropriate. Statistical uncertainty will be expressed in terms of a relative risk and 95% confidence intervals.

## Discussion

Endotracheal intubation remains a challenging procedure, with multiple potential complications such as mucosal edema, ulcerations, or vocal cord injuries [6]. Although most complications are generally reversible, some patients develop severe symptoms [19]. Previous reviews showed the incidence of PES varied widely from 1.5 to 26.3%, and the incidence of reintubation due to laryngeal edema or PES was also quite variable at 1.1–10.5% [6]. While a recent study reported 29% of the patients intubated on an emergency basis had symptoms of stridor on extubation, 45% of these patients who required reintubation had symptoms of post-extubation upper airway obstruction [7], illustrating that PES and extubation failure were much more common in ED patients. Moreover, a study conducted among ED intubated patients showed 35% under multiple intubations, which was associated with a higher incidence of adverse events [20].

The type and dose of prophylactic corticosteroid used varied in adult populations. There were over 10 corticosteroid treatments reported in previous studies, all with different dosing and timings [5]. Although several studies found a multiple dose strategy may be more efficient than a single-dose strategy [1, 18, 21, 22], this is not optimal for ED use due to relative complexity over a single dose. In addition, a multiple dose strategy requires a longer pre-extubation period, which may cause unnecessary delays and increase the risk of complications in the ED setting. Methylprednisolone achieved a peak plasma concentration at about 0.8 h after intravenous administration [11] and possesses a half-life of approximately 2.5 h [23]. The pharmacology of methylprednisolone makes it a good choice for a single-dose, ED-based strategy.

Although few studies reported severe complications associated with prophylactic corticosteroids before extubation [10, 18, 21, 24, 25], a recent study led by Kuriyama [22] reported significantly increased blood glucose levels caused by corticosteroid use, especially among patients with underlying diabetes mellitus. Hyperglycemia, considered as an adaptive-stress response, is frequently present in critically ill patients [26]. A 2-day monitoring for the changes in blood glucose after methylprednisolone treatment will be implemented in our study, with temporary insulin therapy implemented if blood glucose levels increase more than 100 mg/dl.

There are some limitations to this study. First, our study mainly focuses on the initial conditions of intubated patients in the ED. Esteban [27] found that survival in patients receiving mechanical ventilation depended on not only each patient’s condition when initiating mechanical ventilation, but also on how the condition changes and complications develop subsequently. Although we will attempt to control for complications, these may still affect the outcomes. Second, we include extubation failure as one of our secondary outcomes, but it is important to mention that other factors such as pulmonary edema also lead to extubation failure and could be alleviated by corticosteroids as well. Third, there is currently no universally acknowledged extubation standard, so extubation decision made mainly on clinical experience.

This will be the first study looking at the prophylactic methylprednisolone for preventing PES in patients after an emergency intubation. As more patients spend longer periods of time in the ED, it is important to understand the best method for minimizing the complications and maximizing ED patient safety during extubation.

## Trial status

This trial plans to start recruiting patients by the time this article is published. Recruitment is expected to be finished within 10 months.

## Supplementary Information

**Additional file 1.**

## Data Availability

Dataset generated and/or analyzed during the current study are available from the corresponding author (Jun Xu) upon reasonable request.

## Abbreviations

PES: Post-extubation stridor
CLT: Cuff-leak test
GCS: Glasgow Coma Scale
APACHE II: Acute Physiology and Chronic Health
RASS: Richmond Agitation-Sedation Scale
ABGs: Arterial blood gases
SBT: Spontaneous breathing trial
SaO_2_: Arterial blood oxygen saturation
PETCO_2_: End tidal carbon dioxide
BMI: Body mass index
ED: Emergency department
