# Supplementary material for: Efficacy of prophylactic methylprednisolone on reducing the risk of post-extubation stridor in patients after an emergency intubation: study protocol for a randomized controlled trial
Source: Trials. 2021 Jan 6;22:30. doi: 10.1186/s13063-020-04994-9 (PMC7787643; doi:10.1186/s13063-020-04994-9)
Supplement: Supplementary file 1 — Additional file 1. [file 13063_2020_4994_MOESM1_ESM.pdf]

Efficacy of prophylactic methylprednisolone on reducing the risk of post-extubation stridor in patients after an emergency intubation: study protocol for a randomized controlled trial

Jingyi Wang[1#], Joseph Harold Walline[2#], Lu Yin[1], Yili Dai[1], Jiayuan Dai[1], Huadong Zhu[1], Xuezhong Yu[1], Jun Xu[1*]

1 Department of Emergency Medicine, Peking Union Medical College Hospital, Beijing, China

2 Accident and Emergency Medicine Academic Unit, The Chinese University of Hong Kong, Prince of Wales Hospital, Hong Kong, China

First Authors#: Jingyi Wang and Joseph Harold Walline contributed equally to the manuscript.

Jingyi Wang, Email: Jingyiw1996@126.com

Joseph Harold Walline, Email: jwalline@cuhk.edu.hk

Corresponding Author:

Jun Xu, Email: xujunfree@126.com

Abstract

Background: Post-extubation stridor (PES) is one of the most common complications of invasive respiratory support, with severe cases leading to possible extubation failure (reintubation within 48 hours) and increased mortality. Previous studies confirmed that prophylactic corticosteroids play an important role in reducing the risk of PES and extubation failure. However, few studies have looked at the efficacy of corticosteroids on preventing PES in patients after an emergency intubation.

Aim: To evaluate whether a single dose of methylprednisolone given over a set timeframe before extubation is effective in preventing PES in patients after an emergency intubation.

Methods: A multicenter, randomized, placebo-controlled trial will be performed in an emergency department (ED) setting. The trial will include 132 patients who fail a cuff-leak test (CLT) prior to the intervention. Patients will be randomly assigned to either intravenous methylprednisolone (40mg) or placebo four hours prior to extubation. Other eligible patients who pass the CLT will be included in a non-intervention (observation) group. The primary endpoint is the incidence of PES within 48 hours after extubation. Secondary endpoints include oxygen therapy, respiratory support requirements, reintubation secondary to PES, adverse effects within 48 hours after extubation, hospital length of stay and hospital mortality.

Discussion: Patients who are intubated on an emergency basis have a higher risk of intubation-related complications. Previous studies have examined treatment regimens involving more than 10 different variations on corticosteroid treatments for PES

prevention, while for ED therapy only a simple and effective treatment would be appropriate. Corticosteroid administration is usually accompanied by adverse effects, thus this study will be important for further risk stratification among intubated ED patients.

Trial registration: Chictr.org.cn, ChiCTR2000030349. Registered on 29 Feb 2020.

Keywords: Post-extubation stridor, corticosteroid, emergency airway management, multicenter, randomized controlled trial

Background

Endotracheal intubation with mechanical ventilation is a common life support method in emergency resuscitation. However, intubation/extubation and invasive mechanical ventilation may lead to the development of complications such as post-extubation stridor (PES) or extubation failure (defined as reintubation within 48 hours). Both of these complications are associated with a higher risk of ventilator associated pneumonia, prolonged hospital length of stay and increased mortality[1].

Corticosteroids have a broad anti-inflammatory effect which is well described in previous studies[2,3]. They also have a vasopressor effect that contributes to reduced airway edema and less microvascular fluid leakage[4],which may help prevent laryngeal edema and PES . While a large body of evidence has revealed that prophylactic corticosteroids effectively reduce the incidence of PES and reintubation among high-risk patients[5], few studies have evaluated their effectiveness in patients after an emergency intubation. Due to the large ED population and impatient ward shortage, many patients are both intubated and extubated while remaining in the ED setting. Comparing with the previous studies developed in intensive care units(ICUs)[18], Shinohara[6] found the incidence of PES was higher in patients intubated on an emergency basis. This may due to multiple factors, including lack of familiarity with a patient's history, limited time for airway evaluation and management, contraindications to many sedative agents due to an unstable clinical condition (e.g. hypotension or altered mental status), massive intravenous fluid therapy, or patient-endotracheal tube size mismatch.

Ideally, patients at high risk for PES should be identified before extubation. The cuff-leak test (CLT), defined as the difference in the actual tidal volume before and after cuff deflation, has been proposed for this purpose[7, 8]. Previous studies described a cuff leak volume less than 110ml or 24% of tidal volumes as insufficient[9-11], and evidence showed absent or insufficient cuff leak volume suggested an increased risk of PES and extubation failure[7]. Several studies showed the CLT has a good specificity but poor sensitivity, and the accuracy for predicting PES varied with different studies[13-15]. Moreover, the measured tidal volume could also be influenced by other factors such as system compliance or airflow resistance[13]. However, the CLT is still preferred as it has been showed to decrease the risk of PES and reintubation[8].

We conceived the present protocol in order to evaluate whether a single dose of methylprednisolone given over a set timeframe before extubation is effective in reducing the incidence of PES in patients who had been intubated on an emergency basis and were approaching extubation. We hope to answer this question through a multicenter, double-blind, randomized controlled trial.

Methods

Trial design and setting

This is an investigator-initiated, multicenter, superiority, randomized controlled trial that will include 132 participants in intervention arm. Six large tertiary hospitals in China will be involved in this trial. This study was prospectively registered with

Chictr.org.cn on February 29, 2020 (identifier: ChiCTR 2000030349).The Ethics Committee of Peking Union Medical College Hospital, Chinese Academy of Medical Science and Peking Union Medical College in Beijing has approved the trial protocol. The schedule of enrollment, intervention, data collection and assessment follows the Standardized Protocol Items: Recommendations for Interventional Trials (SPIRIT) guidelines (see Figure 1 for checklist and Additional File 1 for more details).

Study population

Patients will be eligible for inclusion if they are aged 18-80 years, admitted into the Emergency Department(ED) and intubated emergently for one of the following conditions: (1)severe traumatic injury, (2)Glasgow Coma Scale (GCS) score<8, (3)hypoventilation, (4)persistent hypoxemia ($SaO2\leq 90\%$) despite supplemental oxygen, (5)persistent hypotension despite vasopressor treatment, or (6)cardiac arrest. Since most participants in our study will have no capacity to make decisions, written informed consent will be acquired from each patient's next of kin prior to inclusion. Patients or their next of kin will be informed of the purpose, procedures, potential risks, and benefits of the study. Participants will be allowed to withdraw from the trial at any time without consequence.

Patients will be excluded if they meet at least one of the following criteria: pregnant or breastfeeding, chronically treated with corticosteroids or other anti-inflammatory drugs, extubated for patient comfort or on family request, unplanned or self-extubation, vocal cord dysfunction, deep sedation (defined as a Richmond Agitation-Sedation Scale (RASS) score≤-4), or those diagnosed with gastrointestinal

hemorrhage within the past three months.

There will be a research coordinator at each hospital to promote and monitor the trial.

Intervention, control and observation groups

Once eligible patients successfully pass a spontaneous breathing trial (SBT), a CLT will be performed to evaluate their risk for developing PES. Patients who had a cuff-leak volume(CLV) less than 24% of vital volume will be included in the intervention arm, where they will be randomly allocated to either the corticosteroid group or the placebo group. The planned extubation will be implemented within six hours after allocation. Patients in the corticosteroid group and placebo group will be treated with an intravenous injection of methylprednisolone 40mg (corticosteroid) or an equivalent volume of isotonic saline (placebo) four hours prior to extubation.

Patients with a CLV >24% of tidal volume will serve as a control group, where extubation will be performed within two hours after CLT completion.

All other standard precautions or treatments will be implemented in all patients. Precautions include gentle extubation to avoid laryngotracheal mucosal injury, strict endotracheal tube (EET) fixation to avoid repeated friction between the EET and laryngotracheal mucosa, EET nursing to reduce contamination risk. Any treatments for PES will be applied in accordance with each patient’s condition, including: oxygen therapy with inhaled corticosteroid for mild airway edema, intravenous administration of hydrocortisone (100mg) for moderate airway edema. Non-invasive ventilation or re-intubation will be carried out as clinically indicated if the above treatments are ineffective.

Study endpoints

The primary endpoint is the occurrence of PES within the first 48 hours after extubation. Secondary endpoints include oxygen therapy after extubation, respiratory support requirements, reintubation secondary to PES within 48 hours after extubation , adverse effects (glycemic change, gastrointestinal bleeding or neuropsychiatric events). Clinical assessments will be done at 5, 15, 30, and 60 minutes, and then at 3, 6, 12, 24, 36 and 48 hours after extubation. Hospital length of stay and hospital mortality will also serve as secondary endpoints. (see Figure 2 for study flow chart).

Sample size calculation

We used a Chi-Squared statistical analysis of minimum sample size required to evaluate the effect of corticosteroids on our study outcomes. Cheng[9] showed that either a single or multiple doses of methylprednisolone six hours before extubation reduced the incidence of PES compared to placebo (11.6%/7.1% vs 30.2%,). Cheng[10] later reported that a single dose of methylprednisolone four hours before extubation significantly reduced the incidence of PES from 39.4% to 15.8%. Other studies treating with prophylactic dexamethasone showed that it decreased the incidence of PES from 27.5-30.2% to 8.8-11.5% [11, 12].We therefore anticipate an incidence of PES of 10% in the treatment group and 30% in the placebo group. With an 80% power to detect a difference and a two-sided alpha of 0.05, we anticipate needing 59 subjects in each group. Assuming a drop-out rate of 10%, we calculate a total minimum sample size of 132 subjects.

Randomization and blinding

As female sex has been reported to be an independent risk factor for stridor and extubation failure [5,6, 9,16,17], a gender-stratified block randomization will be performed in a 1:1 ratio. Within each stratum, a random block size of four will be used. After completion of the pre-extubation assessment and obtaining written informed consent, participants will be randomly assigned to either the intervention group or the control group. Staff not assigned to patient therapy or assessment will be responsible for injecting the intervention, thus both patients and therapists involved will be blinded to treatment allocation. All analyses will be performed on an intention-to-treat basis.

Data collection and management

For each participant, anthropometric data (gender, age, body mass index (BMI)) and baseline characteristics such as GCS score, Acute Physiology and Chronic Health II (APACHE II) score, vital signs (blood pressure, heart rate, respiratory rate, blood oxygen saturation and temperature) and arterial blood gases (ABGs) will be recorded as long as the patients are in-hospital. Arterial blood oxygen saturation (SaO_2), as well as end tidal carbon dioxide ($PETCO_2$) if available, will be continuously recorded. In order not to cause unnecessary pain to patients, ABGs will be collected when deemed clinically necessary during follow-up, while other baseline characteristics will be recorded at set times.

We will also record incidence of the following for each enrolled patient:

1. PES, defined as a presence of an audible high-pitched sound with respiration

2. Oxygen therapy after extubation (e.g. nasal catheter, reservoir mask or venturi mask)
3. Requiring a respiratory support , as defined by:
    a. Presence of respiratory acidosis (an arterial pH of less than 7.35 with a partial pressure of arterial carbon dioxide of more than 45mmHg)
    b. Clinical signs of increased respiratory effort (use of accessory muscles, intercostal retractions, or paradoxical motion of the abdomen)
    c. Respiratory rate >30 breaths/min for two consecutive hours
    d. Hypoxemia (SaO_2 of <90% with an FiO_2 >50%)
4. Reintubation secondary to PES
5. Adverse effects due to corticosteroids
6. Hospital length of stay
7. Hospital mortality.

Clinical data will be collected locally via the Research Electronic Data Capture (REDCap) system, an Internet-based electronic case report form (CRF). The research coordinators at each hospital will form a steering committee, which provides training and reviews study processes, to improve adherence to the protocol and resolve problems. In addition, they will regularly audit CRFs and contact responsible medical staff members every three months to ensure data quality and accuracy. All data use will be limited to study analysis only, and no individuals' personal information will be published. Data confidentiality will be under the supervision of the study coordinators and the Ethics Committee of Peking Union Medical College Hospital.

Statistical methods

The primary endpoint will be analyzed on an intention-to-treat basis, regardless of whether subjects complete their originally allocated treatment study protocol. Any reasons for protocol violations will be recorded and described. All p-values will be two-tailed, and significance will be a p-value <0.05. Data will be presented as frequencies and percentages for categorical variables. Continuous variables will be expressed as means with standard deviations (when normally distributed) or as medians with interquartile ranges (for skewed distribution). Student’s t-test (normal distribution) or Mann-Whitney U test (skewed distribution) will be used for group comparisons. Categorical variables will be compared using Pearson’s chi-squared test or Fisher’s exact test as appropriate. Statistical uncertainty will be expressed in terms of a relative risk and 95% confidence intervals.

Discussion

Endotracheal intubation remains a challenging procedure, with multiple potential complications such as mucosal edema, ulcerations or vocal cord injuries[18]. Although most complications are generally reversible, some patients develop severe symptoms[19]. Previous reviews showed the incidence of PES varied widely from 1.5 % to 26.3 %, and the incidence of reintubation due to laryngeal edema or PES was also quite variable at 1.1–10.5 % [18]. While a recent study reported 29% of the patients intubated on an emergency basis had symptoms of stridor on extubation, 45% of these patients who required reintubation had symptoms of post-extubation upper

airway obstruction[6], illustrating that PES and extubation failure were much more common in ED patients. Moreover, a study conducted among ED intubated patients showed 35% under multiple intubations, which was associated with a higher incidence of adverse events[20].

The type and dose of prophylactic corticosteroid used varied in adult populations. There were over 10 corticosteroid treatments reported in previous studies, all with different dosing and timings[5]. Although several studies found a multiple dose strategy may be more efficient than a single dose strategy [1,17,22,23], this is not optimal for ED use due to relative complexity over a single-dose. In addition, a multiple dose strategy requires a longer pre-extubation period, which may cause unnecessary delays and increase the risk of complications in the ED setting. Methylprednisolone achieved a peak plasma concentration at about 0.8 hours after intravenous administration[10], and possesses a half-life of approximately 2.5 hours[26]. The pharmacology of methylprednisolone makes it a good choice for a single dose, ED-based strategy.

Although few studies reported severe complications associated with prophylactic corticosteroids before extubation [9,17,22,25],a recent study led by Kuriyama [23] reported significantly increased blood glucose levels caused by corticosteroid use, especially among patients with underlying diabetes mellitus. Hyperglycemia, considered as an adaptive-stress response, is frequently present in critically ill patients[24]. A two-day monitoring for the changes in blood glucose after

methylprednisolone treatment will be implemented in our study, with temporary insulin therapy implemented if blood glucose levels increase more than 100mg/dl.

There are some limitations to this study. First, our study mainly focuses on the initial conditions of intubated patients in the ED. Esteban[27] found that survival in patients receiving mechanical ventilation depended on not only each patient's condition when initiating mechanical ventilation, but also on how the condition changes and complications develop subsequently. Although we will attempt to control for complications, these may still affect the outcomes. Second, we include extubation failure as one of our secondary outcomes, but it's important to mention that other factors such as pulmonary edema also lead to extubation failure, and could be alleviated by corticosteroids as well. Third, there is currently no universally acknowledged extubation standard, so extubation decision made mainly on clinical experience.

This will be the first study looking at the prophylactic methylprednisolone for preventing PES in patients after an emergency intubation. As more patients spend longer periods of time in the ED, it is important to understand the best method for minimizing the complications and maximizing ED patient safety during extubation. .

Trial status

This trial plans to start recruiting patients by the time this article is published. Recruitment is expected to be finished within 10 months.

List of Abbreviations

PES: post-extubation stridor; CLT: cuff-leak test; GCS: Glasgow Coma Scale; APACHE II: Acute Physiology and Chronic Health; RASS: Richmond agitation-sedation scale; ABGs: arterial blood gases; SBT: spontaneous breathing trial; SaO_2: arterial blood oxygen saturation; $PETCO_2$: end tidal carbon dioxide; BMI: body mass index; ED: Emergency Department.

Ethical approval and consent to participate

The Ethics Committee of Peking Union Medical College Hospital approved this trial protocol (reference number JS-2334) on April 9, 2020. Informed consent will be obtained from all participants' next of kin.

Consent for publication

Not Applicable.

Availability of data and materials

Dataset generated and/or analyzed during the current study are available from the corresponding author (Jun Xu) upon reasonable request.

Competing interests

The authors declare no competing interest.

Funding

The study is funded by the Chinese Academy of Medical Sciences' Innovation Fund for Medical Sciences (CIFMS, 2017-I2M-1-009). This funding supports the data collection, analysis, and interpretation as well as writing of the manuscript.

Authors' contributions

JyW and JHW drafted the manuscript. LY, YlD and JyD co-authored the writing of the manuscript. JX proposed the research question and helped conceptualize the protocol. HdZ and XzY critically assessed the study design. All authors read and approved the final manuscript.

References

1. McCaffrey J, Farrell C, Whiting P, Dan A, Bagshaw SM, Delaney AP. Corticosteroids to prevent extubation failure: a systematic review and meta-analysis. Intensive Care Med. 2009; doi:10.1007/s00134-009-1473-9

2. Strehl C, Buttgereit F. Optimized glucocorticoid therapy: teaching old drugs new tricks. Mol Cell Endocrinol. 2013; doi:10.1016/j.mce.2013.01.026

3. Rhen T, Cidlowski JA. Antiinflammatory action of glucocorticoids--new mechanisms for old drugs. N Engl J Med. 2005; doi:10.1056/NEJMra050541

4. Beigelman A, Chipps BE, Bacharier LB. Update on the utility of corticosteroids in acute pediatric respiratory disorders. Allergy Asthma Proc. 2015; doi:10.2500/aap.2015.36.3865

5. Kuriyama A, Umakoshi N, Sun R. Prophylactic Corticosteroids for Prevention of Postextubation Stridor and Reintubation in Adults: A Systematic Review and Meta-analysis. Chest. 2017;. doi:10.1016/j.chest.2017.02.017

6. Shinohara M, Iwashita M, Abe T, Takeuchi I. Risk factors associated with symptoms of post-extubation upper airway obstruction in the emergency setting. J Int Med Res. 2020;. doi:10.1177/0300060520926367

7. Ouellette DR, Patel S, Girard TD, et al. Liberation From Mechanical Ventilation in Critically Ill Adults: An Official American College of Chest Physicians/American Thoracic Society Clinical Practice Guideline: Inspiratory Pressure Augmentation During Spontaneous Breathing Trials, Protocols Minimizing Sedation, and Noninvasive Ventilation Immediately After Extubation. Chest. 2017;

doi:10.1016/j.chest.2016.10.036

8. Girard TD, Alhazzani W, Kress JP, et al. An Official American Thoracic Society/American College of Chest Physicians Clinical Practice Guideline: Liberation from Mechanical Ventilation in Critically Ill Adults. Rehabilitation Protocols, Ventilator Liberation Protocols, and Cuff Leak Tests. Am J Respir Crit Care Med. 2017; doi:10.1164/rccm.201610-2075ST

9. Cheng KC, Hou CC, Huang HC, Lin SC, Zhang H. Intravenous injection of methylprednisolone reduces the incidence of postextubation stridor in intensive care unit patients [published correction appears in Crit Care Med. 2007 May;35(5):1454]. Crit Care Med. 2006; doi:10.1097/01.CCM.0000214678.92134.BD

10. Cheng KC, Chen CM, Tan CK, Chen HM, Lu CL, Zhang H. Methylprednisolone reduces the rates of postextubation stridor and reintubation associated with attenuated cytokine responses in critically ill patients. Minerva Anestesiol. 2011;77(5):503-509.

11. Lee CH, Peng MJ, Wu CL. Dexamethasone to prevent postextubation airway obstruction in adults: a prospective, randomized, double-blind, placebo-controlled study. Crit Care. 2007; doi:10.1186/cc5957

12. Yu Y, Zhu C, Mao E, et al. Use of dexamethasone for preventing postextubation airway obstruction in adults: a prospective randomized study. J Intern Med Concepts Pract. 2014;9(2): 134-137

13. De Backer D. The cuff-leak test: what are we measuring? Crit Care. 2005;. doi:10.1186/cc3031

14. Schnell D, Planquette B, Berger A, et al. Cuff Leak Test for the Diagnosis of Post-Extubation Stridor: A Multicenter Evaluation Study. J Intensive Care Med. 2019; doi:10.1177/0885066617700095

15. Zhou T, Zhang HP, Chen WW, et al. Cuff-leak test for predicting postextubation airway complications: a systematic review. J Evid Based Med. 2011; doi:10.1111/j.1756-5391.2011.01160.x

16. Ho LI, Harn HJ, Lien TC, Hu PY, Wang JH. Postextubation laryngeal edema in adults. Risk factor evaluation and prevention by hydrocortisone. Intensive Care Med. 1996; doi:10.1007/BF02044118

17. François B, Bellissant E, Gissot V, et al. 12-h pretreatment with methylprednisolone versus placebo for prevention of postextubation laryngeal oedema: a randomised double-blind trial. Lancet. 2007; doi:10.1016/S0140-6736(07)60526-1

18. Pluijms WA, van Mook WN, Wittekamp BH, Bergmans DC. Postextubation laryngeal edema and stridor resulting in respiratory failure in critically ill adult patients: updated review. Crit Care. 2015; doi:10.1186/s13054-015-1018-2

19. Wittekamp BH, van Mook WN, Tjan DH, Zwaveling JH, Bergmans DC. Clinical review: post-extubation laryngeal edema and extubation failure in critically ill adult patients. Crit Care. 2009; doi:10.1186/cc8142

20. Yamanaka S, Goldman RD, Goto T, Hayashi H. Multiple intubation attempts in the emergency department and in-hospital mortality: A retrospective observational study. Am J Emerg Med. 2020; doi:10.1016/j.ajem.2019.06.028

21. Fan T, Wang G, Mao B, et al. Prophylactic administration of parenteral steroids

for preventing airway complications after extubation in adults: meta-analysis of randomised placebo controlled trials. BMJ. 2008; Published 2008 Oct 20. doi:10.1136/bmj.a1841

22. Khemani RG, Randolph A, Markovitz B. Corticosteroids for the prevention and treatment of post-extubation stridor in neonates, children and adults. Cochrane Database Syst Rev. 2009; doi:10.1002/14651858.CD001000.pub3

23. Kuriyama A, Egawa S, Kataoka J, Sakuraya M, Matsumura M. Adverse events associated with prophylactic corticosteroid use before extubation: a cohort study. Ann Transl Med. 2020; doi:10.21037/atm-20-1790

24. Godinjak A, Iglica A, Burekovic A, et al. Hyperglycemia in Critically Ill Patients: Management and Prognosis. Med Arch. 2015; doi:10.5455/medarh.2015.69.157-160

25. Jaber S, Chanques G, Matecki S, et al. Post-extubation stridor in intensive care unit patients. Risk factors evaluation and importance of the cuff-leak test. Intensive Care Med. 2003; doi:10.1007/s00134-002-1563-4

26. Xiaohui L. Adrenocortical hormones. In: Baofeng Y, Dingfeng S, et al. editors. Pharmacology, 9th edition. People’s Medical Publishing House;2013. p.327-336

27. Esteban A, Anzueto A, Frutos F, et al. Characteristics and outcomes in adult patients receiving mechanical ventilation: a 28-day international study. JAMA. 2002; doi:10.1001/jama.287.3.345

Figures

	STUDY PERIOD														
	Pre-randomization		Primary study period												secondary study period
TIMEPOINT	enrollment	allocation	-4h	0h	5min	15min	30min	1h	3h	6h	12h	24h	36h	48h	Hospital discharge
**ENROLLMENT**															
-Eligibility screen	×														
-Informed consent	×														
-Allocation		×													
**INTERENTIONS**															
-Methylprednisolone therapy			×												
-Placebo therapy			×												
**EXTUBATION**				×											
**DATA COLLECTION AND ASSESSMENT**															
Anthropometric data	×														
*ABGs	×														
Baseline variables	×		×	×	×	×	×	×	×	×	×	×	×	×	
Primary endpoint					×	×	×	×	×	×	×	×	×	×	
Secondary endpoints(a)				×	×	×	×	×	×	×	×	×	×	×	
Continuous points			◆	—	—	—	—	—	—	—	—	—	—	◆	
Secondary endpoints(b)															×

*Figure 1 - Standard Protocol Items: Recommendations for Interventional Trials (SPIRIT) schedule of enrolment, intervention and assessments. Anthropometric data: age, gender, body mass index (BMI).*ABGs: collected for necessity only. Baseline variables: GCS score, APACHE II score, vital signs. Primary endpoint: occurrence of PES. Secondary endpoints (a): postextubation oxygen therapy, respiratory support requirement, reintubation secondary to PES, adverse effects. Secondary endpoints (b): hospital length of stay and hospital mortality. Continuous points: SaO_2 and $PETCO_2$.*

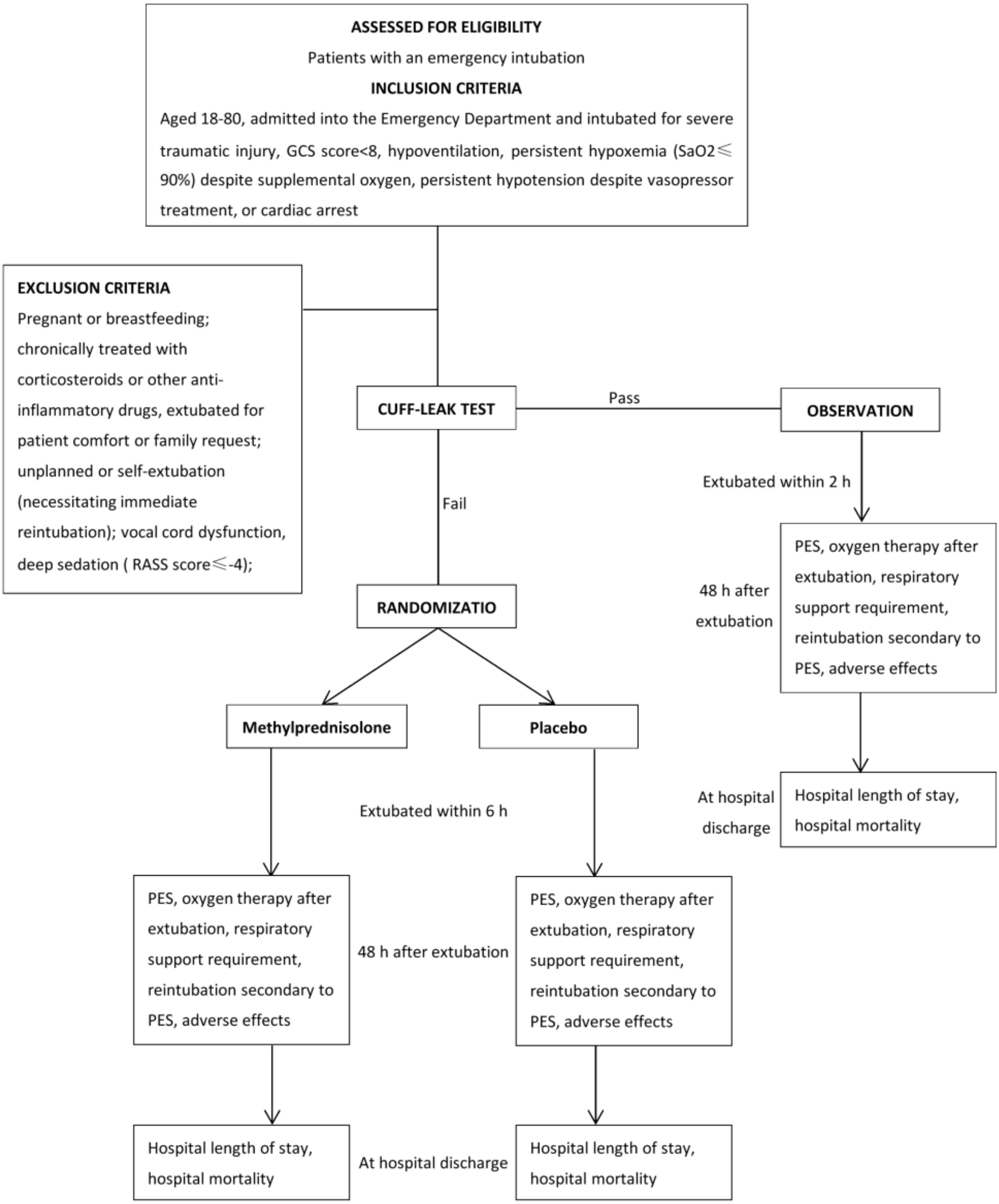

Figure 2-Study flow chart.
